# Do Multiwell Plate High Throughput Assays Measure Loss of Cell Viability Following Exposure to Genotoxic Agents?

**DOI:** 10.3390/ijms18081679

**Published:** 2017-08-02

**Authors:** Razmik Mirzayans, Bonnie Andrais, David Murray

**Affiliations:** Department of Oncology, University of Alberta, Cross Cancer Institute, Edmonton, AB T6G 1Z2, Canada; bonnie.andrais@ahs.ca (B.A.); david.murray5@ahs.ca (D.M.)

**Keywords:** cisplatin, p53 signaling, premature senescence, viability, proliferation, MTT, XTT, CellTiter-Blue

## Abstract

Cell-based assays in multiwell plates are widely used for radiosensitivity and chemosensitivity assessment with different mammalian cell types. Despite their relative ease of performance, such assays lack specificity as they do not distinguish between the cytostatic (reversible/sustained growth arrest) and cytotoxic (loss of viability) effects of genotoxic agents. We recently reported studies with solid tumor-derived cell lines demonstrating that radiosensitivity as measured by multiwell plate colorimetric (e.g., XTT) and fluorimetric (e.g., CellTiter-Blue) assays reflects growth arrest but not loss of viability. Herein we report similar observations with cancer cell lines expressing wild-type p53 (A549 lung carcinoma) or mutant p53 (MDA–MB-231 breast carcinoma) after treatment with the chemotherapeutic drug cisplatin. Importantly, we show that treatment of cancer cells with concentrations of cisplatin that result in 50% effect (i.e., IC_50_) in multiwell plate assays trigger the emergence of growth-arrested cells that exhibit highly enlarged morphology, remain viable and adherent to the culture dish, and metabolize the tetrazolium salt 3-(4,5-dimethylthiazol-2-yl)-2,5-diphenyl-tetrazolium bromide (MTT) to its formazan derivative. The emergence of markedly enlarged viable cells complicates the interpretation of chemosensitivity data obtained with multiwell plate high throughput assays. Relying solely on IC_50_ values could be misleading.

## 1. Introduction

The chemotherapeutic drug *cis*-diamminedichloroplatinum (II) (cisplatin) induces cytotoxicity in various cell types when administered at very high concentrations (reviewed in [1,2]). However, like other genotoxic agents, when administered at moderate, clinically relevant concentrations, cisplatin triggers a sustained growth-arrested response (and not cell death) in human solid tumor-derived cell lines [1,2]. The growth-arrested response predominantly reflects stress-induced premature senescence (SIPS) and/or the development of polyploid/multinucleated giant cells, depending on the status of p53, its downstream effector p21^WAF1^ (p21), and other factors [1,2]. Such growth-arrested cancer cells exhibit highly enlarged morphology, remain viable for long times (weeks) post-treatment, and secrete a myriad of biologically active factors [1,2,3,4,5,6,7,8].

The observation that polyploid/multinucleated giant cells remain viable and secrete growth-promoting factors was first reported by Puck and Marcus over 60 years ago [9]. In addition to secreting such factors, numerous studies reported in the past decade have demonstrated that giant cells that emerge in solid tumors in response to therapeutic agents can contribute to cancer relapse by giving rise to progeny with stem cell-like properties [10,11,12,13,14,15,16,17,18,19,20,21,22,23,24,25,26,27,28]. Giant cells can give rise to tumor-repopulating cells through nuclear budding or bursting, similar to simple organisms such as fungi, as well as through depolyploidization, involving key mediators of meiosis, self-renewal, and mitosis [1,2]. Disconcertingly, only a single multinucleated giant cell has been reported to be sufficient to generate metastatic tumors in a mouse model [14].

The emergence of highly enlarged cells following genotoxic stress complicates the interpretation of radiosensitivity and chemosensitivity data obtained by cell-based assays that are performed in a multiwell plate format. As extensively discussed recently [1,2,29,30], despite their ease of use, all multiwell plate assays lack specificity as they measure the sum of transient cell cycle checkpoint activation (pro-survival), growth arrest that may or may not be reversible, and cell death. Unfortunately, the results obtained with such assays have often been misinterpreted to reflect loss of viability and hence cell death.

We recently reported studies demonstrating that human solid tumor-derived cell lines with differing p53/p21 status respond to moderate doses of ionizing radiation (e.g., 8 Gy) by exhibiting sustained growth arrest but not apoptosis or other modes of cell death, and that this growth-arrested response is attributable to the emergence of highly enlarged cells [29,30]. In addition, virtually all enlarged cells that remain adherent to the culture dish for long times (weeks) post-irradiation retain membrane integrity and exhibit the ability to metabolize the tetrazolium salt 3-(4,5-dimethylthiazol-2-yl)-2,5-diphenyl-tetrazolium bromide (MTT) to its water-insoluble formazan derivative [29,30]. Accordingly, we concluded that, for studies that do not require high throughput screening, single-cell observation methods (single-cell MTT) provide a more reliable tool for determining cell viability/metabolic activity as compared to colorimetric/fluorimetric measurements performed in a multi-well format [29].

The purpose of the current study was to determine the impact of cancer cell enlargement on chemosensitivity as measured by multiwell plate colorimetric (XTT (2,3-bis-(2-methoxy-4-nitro-5-sulfophenyl)-2*H*-tetrazolium-5-carboxanilid)) and fluorimetric (CellTiter-Blue) assays in the human cancer cell lines A549 (lung carcinoma) and MDA–MB-231 (breast carcinoma), which express wild-type and mutant p53, respectively. We show that treatment of both cell lines with concentrations of cisplatin that result in 50% inhibitory effect (IC_50_) in multiwell plate assays does not induce cell death as evaluated by the trypan blue-exclusion assay, but instead triggers growth arrest through the emergence of highly enlarged cells that remain adherent to the culture dish for the duration of the experiments (3 days continuous drug exposure). Single cell evaluations demonstrated that virtually all cisplatin-treated cells (encompassing highly enlarged cells) metabolize MTT. Thus, genotoxicity assessed in a population of cells by multiwell plate assays does not reflect loss of viability and cell death, at least in the human solid tumor-derived cell lines examined by us. To refer to such assays as informing on “viability” or “cytotoxicity” could be misleading.

## 2. Results

Over three decades ago, we observed that the short-term (3 days) growth inhibition assay involving direct cell counting appeared to be as informative as the long-term (10–14 days) but laborious colony formation assay for genotoxicity assessment with cultured human cells [31,32]. We have recently confirmed this observation with a panel of human solid tumor-derived cell lines after exposure to ionizing radiation [29,30]. An important advantage of our improved growth inhibition assay over the colony formation and colorimetric/fluorimetric assays is that it also enables the investigator to utilize microscopic examination to track the long-term fate of adherent cells following genotoxic treatment. To this end, cells in one set of dishes can be evaluated, either at the time of cell counting (i.e., 3 days post-treatment) or after further incubation, not only for their morphology, but also for responses such as viability, metabolic activity, and genome integrity using a battery of single-cell methods. We assess cell viability by the trypan blue-exclusion assay under conditions that minimize the creation of false positives (e.g., cell detachment by exposure to trypsin) [29,30,33]. We also assess cell metabolic activity by the single-cell MTT assay [29,30]. The advantage of the single-cell MTT assay over the widely-used multiwell assay is that it enables the application of image analysis to determine the metabolic activity of individual cells irrespective of their size.

In the current study we used this approach together with conventional multiwell plate XTT (2,3-bis-(2-methoxy-4-nitro-5-sulfophenyl)-2*H*-tetrazolium-5-carboxanilid) and CellTiter-Blue assays to determine the sensitivity of A549 lung carcinoma and MDA–MB-321 breast carcinoma cell lines to moderate, clinically relevant, concentrations of cisplatin (see below). Details of the XTT (colorimetric) and CellTiter-Blue (fluorimetric) assays were described recently [30]. Such assays provide an indirect measure of proliferation, estimated from the change of absorbance (or fluorescence) of the medium overlaying the cells, using a plate reader. In the XTT assay, for example, the change of color of the medium from yellow (e.g., in no-cell “blank” control wells) to orange (in wells containing cells) is proportional to the number of viable cells per well. It should be noted that, unlike the MTT formazan metabolite which is water insoluble and can be visualized as purple intracellular granules and crystals under a light microscope, the XTT formazan metabolite is water soluble and orange in color [30].

Cisplatin is known to induce a significant degree of apoptosis in many cell types, but only when administered at high concentrations (>20 μM) [1,2]. This is consistent with the observation that cisplatin-induced apoptosis primarily reflects injury to mitochondria rather than to nuclear DNA [34]. Puig et al. [26] used a rat colon carcinoma model and demonstrated that such high, apoptosis-triggering, concentrations of cisplatin cannot be achieved in vivo because of major toxic side effects. When administered at tolerated concentrations, cisplatin induced tumor cell dormancy through the formation of polyploid/multinucleated giant cells. Physiological concentrations of cisplatin, relevant to clinical practice and pharmacological characteristics of the drug measured in tumor and plasma, corresponds to ≤10 μM in cell culture experiments [26].

**Figure 1:**
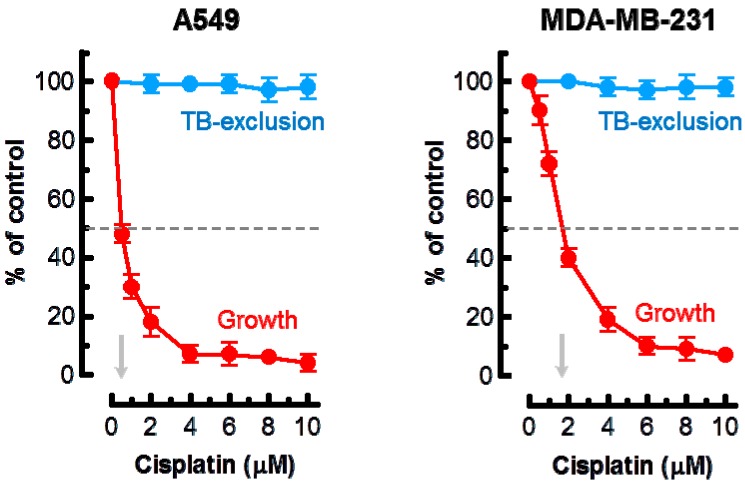
Effect of cisplatin treatment (3 days) on the extent of cell growth and viability of the indicated cell lines, determined by direct cell counting and the trypan blue-exclusion assay, respectively. Arrows show cisplatin concentrations resulting in 50% inhibition of growth. Bars, standard error (SE). TB, trypan blue.

Based on these observations, the highest concentration of cisplatin used in the current experiments was 10 μM. The results of growth inhibition and trypan blue-exclusion assays are presented in Figure 1. In the growth inhibition assay, we determined the cell inoculum size (number of adherent cells per dish at the beginning of genotoxic treatment) and subtracted this number from the number of cells per dish measured after genotoxic treatment (or sham treatment). Under these conditions, ~50% growth inhibition was observed in cultures of the A549 and MDA–MB-231 cell lines after treatment with ~0.5 and ~2 μM cisplatin, respectively. Incubation of these cultures with ≤10 μM cisplatin for 3 days did not result in cell detachment (data not shown) or loss of cell membrane integrity as judged by the trypan blue-exclusion assay (Figure 1).

**Figure 2:**
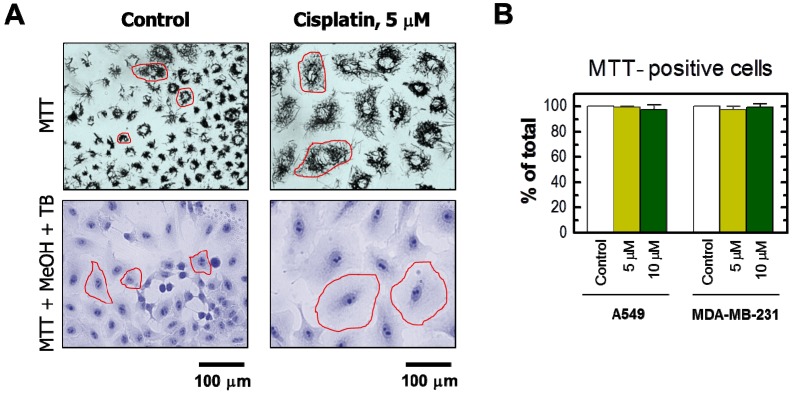
(**A**) Representative bright-field microscopy images depicting the metabolic activity of A549 cells that were incubated with 0 μM (control) or 5 μM cisplatin for 3 days. Metabolic activity was measured by the ability of the cells to convert the yellow MTT to its purple formazan metabolite, which appears as dark granules and crystals. Upper row: images were acquired after incubation of cells with MTT for ~1 h. Lower row: images were acquired after incubating cells with MTT (~1 h), fixing them in methanol (MeOH) for 0.5 min to dissolve the MTT metabolite, removing the resulting purple medium, and mildly staining the fixed cells with trypan blue (TB) to visualize their morphology. All images were acquired at the same magnification. The border of some cells is marked in red for clarity. Images acquired for the MDA–MB-231 cell line were virtually identical to those shown for A549 (also see Figure 2A); (**B**) percentages of MTT-positive cells after the same treatments for both A549 and MDA–MB-231 cell lines. Bars, SE.

**Figure 3:**
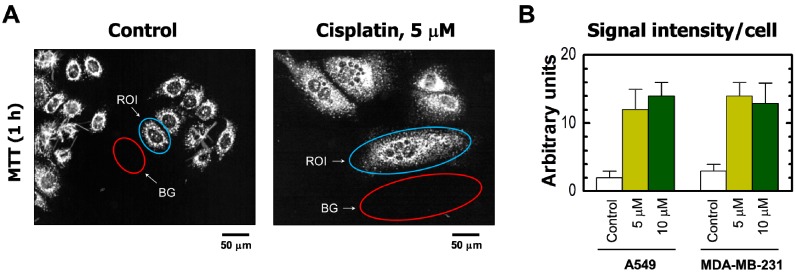
(**A**) Representative images of MDA–MB-231 cells used for image analysis. The images of MTT metabolites were acquired as described in Figure 2 legend (upper row). The images were then converted to grayscale and inverted. Blue and red ovals mark some regions of interest (reflecting MTT metabolites) and corresponding background regions used for image analysis, respectively; (**B**) densitometric evaluation of MTT metabolic activity for the indicated cultures, expressed as signal intensity for regions of interest (cells) after corresponding background corrections. Mean values for at least 30 cells are presented for each sample. Bars, SE. ROI, region of interest; BG, background.

The results obtained by the single-cell MTT assay are presented in Figure 2 and Figure 3. Cisplatin treatment of these cultures resulted in the development of highly enlarged cells that metabolized MTT to intracellular formazan granules and needle-like formazan crystals (Figure 2A and Figure 3A). In response to 5 or 10 μM cisplatin treatment, virtually all cells exhibited enlarged morphology and metabolized MTT (Figure 2). In addition, the level of metabolic activity per cell (estimated from signal intensity in the “region of interest”) was markedly greater for cisplatin-treated cultures than in sham-treated controls (Figure 3).

**Figure 4:**
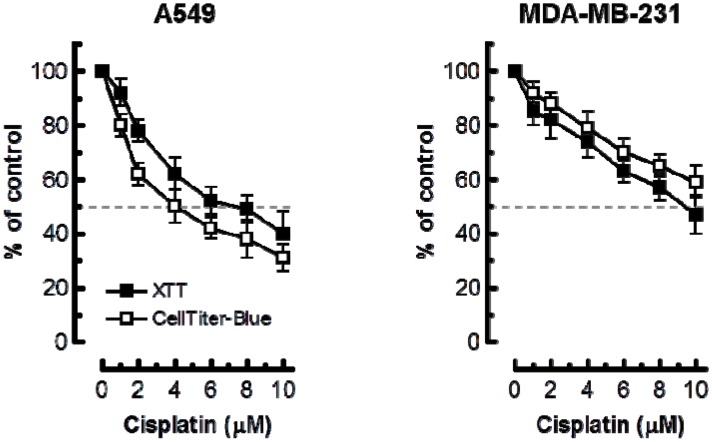
Cisplatin sensitivity of the indicated cell lines evaluated by the 96-well plate XTT (solid squares) and CellTiter-Blue (open squares) assays. Bars, SE.

Considering these observations, we anticipated that the multiwell plate metabolic-based assays would markedly underestimate cisplatin sensitivity as compared to the growth-inhibition assay. The results presented in Figure 4 support this notion. In A549 cells, 50% inhibitory effect required ≥4 μM cisplatin in XTT and CellTiter-Blue assays as compared to ~0.5 μM in the growth-inhibition assay. In MDA–MD-231 cells, 50% effect required ≥10 μM cisplatin in multiwell plate assays as compared to ~2 μM in the growth-inhibition assay.

## 3. Discussion

Many different in vitro methods are available for assessment of radiosensitivity and chemosensitivity in cultured mammalian cells. The most commonly-used methods can be divided into one of the three categories: (i) the long-term (10–14 days) colony formation assay, developed by Puck and Marcus over 60 years ago [9]; (ii) the short-term (3 days) growth inhibition assay involving direct cell counting, used by us [31,32] and others [35,36] since the early 1980s; and (iii) more recently-developed metabolic activity assays that are performed in a multiwell plate format and determine cell proliferation indirectly from the change in absorbance (or fluorescence) of the medium overlaying the cells. The colony formation assay is performed with cells plated out at very low densities in a large volume of medium, whereas the growth-inhibition and multiwell plate assays are performed with a population of cells under conditions that facilitate some aspects of intercellular communication through both gap junctions and diffusible factors [33]. Among these, multiwell plate assays are indispensable for high throughput screening purposes [37,38] despite their several shortcomings [29,30,37,39,40,41,42,43,44].

As discussed recently [1,2], the effect measured by these cell-based assays was originally believed to reflect loss of viability. In our earlier studies, we assumed radiosensitivity as measured by the 96-well plate XTT assay to be associated with early apoptosis and necrosis [33,45]. Many authors, as well as some manufacturers of multiwell plate assays, continue to make such assumptions. The rationale behind this interpretation is that genotoxic stress has been assumed to either activate cell cycle checkpoints to facilitate repair and promote survival, or to trigger cell demise through apoptosis and other modes of cell death. Thus, stress-induced extended growth arrest has been considered to reflect loss of viability and cell death. Accordingly, statements to the effect that “the MTT (or XTT, CellTiter-Blue, etc.) cell proliferation assay was used to determine loss of cell viability and cytotoxicity” appear in numerous reports (too many to cite).

In retrospect, an important observation reported by Puck and Marcus in 1956 has been largely overlooked. Using the human HeLa cervical carcinoma cell line, these authors demonstrated that radiation exposure resulted in the development of highly enlarged cells that contained multiple nuclei, ceased to proliferate (or divided very slowly), remained viable and secreted factors with growth stimulating properties [9]. We now know that exposure of solid tumor-derived cell lines to moderate, clinically relevant, doses of cancer therapeutic agents results in the development of highly enlarged growth-arrested cells that remain viable and can contribute to cancer recurrence not only by secreting growth-promoting factors but also by giving rise to tumor-repopulating progeny through depolyploidization, involving meiosis, mitosis and self-renewal genes, as well as the process of nuclear budding [10,11,12,13,14,15,16,17,18,19,20,21,22,23,24,25,26,27,28]. Thus, therapy-induced extended growth arrest can reflect a mechanism of survival and not necessarily death for cells within solid tumors [1,2,46].

The potential for cell-based assays to generate misleading information in the context of anticancer drug development has been reviewed by the Nomenclature Committee on Cell Death [47], Husmann [48], Eastman [49], and us [1,2]. We have been interested in determining whether multiwell plate high throughput assays measure loss of viability following genotoxic treatment, as claimed by some manufacturers of such reagents. We recently reported a series of radiosensitivity studies with human solid tumor-derived cell lines with differing p53 status and demonstrated that moderate doses of ionizing radiation (between 2 and 8 Gy) primarily triggered extended growth arrest rather than cytotoxicity [29,30]. Such growth-arrested cancer cells exhibited highly enlarged morphology, retained membrane integrity, and metabolized MTT to its water insoluble formazan derivative. In addition, radiosensitivity as measured by multiwell plate assays was markedly skewed towards resistance when compared to that measured by the growth inhibition assay [29,30]. In the current study we have demonstrated a similar phenomenon for the A549 lung carcinoma cell line (expressing wild-type p53) and the MDA–MB-231 breast carcinoma cell line (expressing mutant p53) after treatment with clinically relevant concentrations of cisplatin (≤10 μM). In addition, the single cell MTT assay demonstrated that the metabolic activity per cell was actually much greater for cisplatin-treated cultures than for sham-treated controls.

We are in the process of evaluating additional cell lines, including the HCT116 colon carcinoma line and its p53 knockout and p21 knockout derivatives, after treatment with moderate concentrations of cisplatin, oxaliplatin, and paclitaxel. The single cell MTT assay has shown that the emergence of highly enlarged, viable, and metabolically active cells is the most overt response of solid tumor-derived cell lines irrespective of their p53/p21 status and the type of chemotherapeutic agent used (unpublished observations). Determining the molecular basis for the apoptosis-resistant phenotype of highly enlarged cancer cells might lead to novel approaches for preventing recurrent disease subsequent to conventional radiotherapy and chemotherapy.

In short, the answer to the question posed in this study appears to be very much negative. Not only do multiwell plate assays not measure loss of viability and cytotoxicity in response to moderate doses of genotoxic agents, they also markedly underestimate the degree of proliferation block as a result of the emergence of highly enlarged cells that remain viable and metabolically active. While extremely valuable for screening purposes, multiwell plate assays can be highly misleading in the absence of more detailed analyses of parameters such as cell viability and metabolic activity in individual cells under conditions that do not create false positives.

## 4. Materials and Methods 

### 4.1. Cells and Culture Conditions

The two cancer cell lines used in the present study were purchased from the American Type Culture Collection (Rockville, MD, USA). Cells were cultured as monolayers in DMEM/F12 nutrient medium supplemented with 5% (*v*/*v*) fetal bovine serum as described [33]. All cultures were free of *Mycoplasma* contamination. 

### 4.2. Reagents

The vital dye trypan blue (Sigma, St. Louis, MO, USA), the CellTiter-Blue reagent (Promega, Madison, WI, USA), and the tetrazolium dyes MTT and XTT (Roche Diagnostics, Penzberg, Germany) were used as recommended by the manufacturers. Cisplatin was purchased from Mayne Pharma (Kirkland, WA, Canada).

### 4.3. Growth Inhibition Assay

Cells were plated in 60-mm dishes (10^5^ cells/5 mL medium/dish) and incubated overnight. The medium was replaced with medium containing different concentrations of cisplatin. After incubation for 3 days, adherent cells were harvested by the use of trypsin and counted by a cell counter (Coulter, Hialeah, FL, USA). The cell inoculum size (number of adherent cells per dish measured just prior to incubation with cisplatin) was also determined; this number was subtracted from the number of cells per dish measured 3 days after cisplatin treatment (or sham treatment). Growth inhibition curves were generated by plotting the extent of cell growth in cisplatin-treated dishes (expressed as a percentage of control cells in sham-treated dishes) as a function of cisplatin concentration.

### 4.4. Multiwell Plate Assays

The XTT (Roche Diagnostics) and CellTiter-Blue (Promega) cell-proliferation assays were performed according to the instructions provided by the suppliers. Briefly, cells were plated in 96-well tissue culture plates at a density of 2000 cells per well in 200 μL of medium and incubated overnight. The medium was replaced with medium (200 μL/well) containing different concentrations of cisplatin. The cells were treated with cisplatin for 3 days. For the XTT proliferation assay, the medium containing cisplatin was removed and the cells were incubated with medium containing XTT and the electron-coupling reagent for 3 h. Absorbance of the wells was determined at 492-nm using the Fluostar Optima FL plate reader (BMG Labtech, Ortenberg, Germany). For the CellTiter-Blue proliferation assay, the cisplatin-containing medium was replaced with medium supplemented with the CellTiter-Blue reagent. After incubation for 3 h, the fluorescence of the medium was measured using the Fluostar Optima FL plate reader. Pilot experiments indicated that, for both cell lines used in the current study, seeding 2000 cell/well and incubating the cells with either the XTT or the CellTiter-Blue reagent for 3 h after cisplatin treatment were optimal conditions. 

## 5. Conclusions

Since 1956, an overwhelming number of articles have established the significance of cancer cell enlargement (reflecting SIPS and/or polyploid/multinucleated giant cells) as a potential mechanism of tumor cell survival and thus therapy failure (reviewed in [1,2]; also see [10,11,12,13,14,15,16,17,18,19,20,21,22,23,24,25,26,27,28]). Unfortunately, cancer cell enlargement has been ignored by numerous studies which have focused on apoptosis as the major response of cancer cells following genotoxic treatment. In fact, cancer cell enlargement is not even mentioned in recent cell death-related review articles and guidelines. Perhaps this is in part because most studies have relied on assays such as immunoblotting, colony formation, and multiwell plate proliferation that measure responses in a population of cells rather than in individual cells. Furthermore, the response measured by multiwell plate assays is often misinterpreted to reflect loss of viability. We have provided clear evidence that: (i) chemosensitivity as measured by multiwell plate assays after treatment with clinically relevant concentrations of cisplatin is not associated with loss of viability; and (ii) such assays underestimate the degree of proliferation block following cisplatin treatment as a result of the emergence of highly enlarged cells that exhibit a much greater metabolic activity than untreated cells.

The growth inhibition coupled with single-cell MTT protocol used herein and in our previous studies [29,30] circumvents the many pitfalls associated with multiwell plate genotoxicity assays. Importantly, our growth-inhibition protocol enables the long-term tracking of the fate of growth-arrested cancer cells subsequent to therapeutic exposures.

However, as pointed out previously [29], “irrespective of the type of assay employed, the take-home message of a large body of data generated in recent decades, including the results presented here, is not different from what Puck and Marcus reported for the HeLa cervical carcinoma cell line 60 years ago. Namely, genotoxic treatment predominantly triggers cancer cell dormancy rather than lethality”. A single dormant (multinucleated giant) cancer cell can in fact be sufficient to generate metastatic tumors in animal models [14]. Thus, a proportion of treated cells that are scored as “dead” in cell-based assays appear to be responsible for cancer recurrence and hence therapy failure.

## Abbreviations

SIPS	Stress-induced premature senescence
MTT	3-(4,5-Dimethylthiazol-2-yl)-2,5-diphenyl-tetrazolium bromide
XTT	2,3-Bis-(2-methoxy-4-nitro-5-sulfophenyl)-2*H*-tetrazolium-5-carboxanilid
CTB	CellTiter Blue
IC_50_	Inhibiting concentration, 50%
SE	Standard error
TB	Trypan blue
ROI	Region of interest
BG	Background
